# B Cell Therapy in Systemic Lupus Erythematosus: From Rationale to Clinical Practice

**DOI:** 10.3389/fmed.2020.00316

**Published:** 2020-07-09

**Authors:** Ioannis Parodis, Marit Stockfelt, Christopher Sjöwall

**Affiliations:** ^1^Division of Rheumatology, Department of Medicine Solna, Karolinska Institutet, Stockholm, Sweden; ^2^Rheumatology, Karolinska University Hospital, Stockholm, Sweden; ^3^Department of Rheumatology and Inflammation Research, Institute of Medicine, Sahlgrenska Academy at the University of Gothenburg, Gothenburg, Sweden; ^4^Division of Inflammation and Infection, Department of Biomedical and Clinical Sciences, Linköping University, Linköping, Sweden

**Keywords:** B cells, systemic lupus erythematosus, therapy, biologics, plasma cells, plasmablasts, lupus nephritis

## Abstract

B cell hyperactivity and breach of tolerance constitute hallmarks of systemic lupus erythematosus (SLE). The heterogeneity of disease manifestations and relatively rare prevalence of SLE have posed difficulties in trial design and contributed to a slow pace for drug development. The anti-BAFF monoclonal antibody belimumab is still the sole targeted therapy licensed for SLE, lending credence to the widely accepted notion that B cells play central roles in lupus pathogenesis. However, more therapeutic agents directed toward B cells or B cell-related pathways are used off-label or have been trialed in SLE. The anti-CD20 monoclonal antibody rituximab has been used to treat refractory SLE during the last two decades, and the anti-type I IFN receptor anifrolumab is currently awaiting approval after one phase III clinical trial which met its primary endpoint and one phase III trial which met key secondary endpoints. While the latter does not directly affect the maturation and antibody production activity of B cells, it is expected to affect the contribution of B cells in proinflammatory cytokine excretion. The proteasome inhibitor bortezomib, primarily directed toward the plasma cells, has been used in few severe cases as an escape regimen. Collectively, current clinical experience and primary results of ongoing clinical trials prophesy that B cell therapies of selective targets will have an established place in the future personalized therapeutic management of lupus patients.

## Introduction

Systemic lupus erythematosus (SLE) is a chronic autoimmune disease that can affect multiple organ systems (1). The treatment of SLE has traditionally been non-specific, with antimalarial agents as the therapeutic cornerstone due to the wide variety of beneficial effects associated with their use (2, 3), and broad immunosuppression being used to hamper the inflammatory state and protect against end-organ damage accrual (4–6). Several of the medications used to treat patients with SLE still have not received approval by regulatory drug agencies. Following the timeline of drug development in the field of rheumatology at large, the development of new therapies for SLE has been hampered due to several reasons.

First, the pronounced heterogeneity of clinical phenotypes poses challenges in developing outcome measures which unanimously and reliably capture response to treatment regarding activity in the afflicted organs, and which also reflect the global SLE disease activity. As a result, the lack of reliable measures for treatment evaluation makes it challenging to design clinical trials to assess drug efficacy. Recruitment of participants has been slow and inadequate in organ-specific trials, whereas the applicability of currently available outcome measures has been questioned in clinically heterogeneous study populations. Borrowed from e.g., rheumatoid arthritis (RA), the treat-to-target concept has also gained attention in SLE (7), and composite measures have been developed to serve as tools for assessing clinical improvement. The SLE Responder Index (SRI) (8) was initially designed to serve as an outcome measure in clinical trials of belimumab (9–11), and the British Isles Lupus Assessment Group (BILAG)-based combined lupus assessment (BICLA) was first used in a phase IIb clinical trial of epratuzumab (12). They were both developed to reflect improvement in SLE disease activity. Other composite tools have been developed to reflect low disease activity, e.g., the Lupus Low Disease Activity State (LLDAS) (13), or remission, e.g., the Definitions of Remission in SLE (DORIS) (14). Both LLDAS and DORIS were designed to be applicable on specific evaluation occasions, and are independent of preceding degree of activity.

Using such tools, the first successful trials (10, 11) resulted in the approval of the first biological agent for the treatment of SLE about one decade ago (15). This agent was belimumab, a monoclonal antibody against the B cell activating cytokine BAFF, further discussed later, and the target was no other than B cells of early maturation stages, lending credence to the historical notion that they have a central role in lupus pathogenesis (16). Indeed, even before the official approval of belimumab as a treatment option, several therapies targeting B cells at different developmental stages have been used off-label (17). This review summarizes the rationale and clinical application of the B cell therapy panorama in SLE.

## B Cells in SLE

The complex SLE disease is characterized by loss of self-tolerance, which leads to immune responses toward endogenous nuclear and cytoplasmic material. In response to these autoantigens, clones of plasma cells produce autoantibodies, which are considered a hallmark of the disease. Autoantibodies may induce inflammation through the formation of immune complexes and through activation of Fc-γ receptors. Arguing for a pathogenic role, autoantibodies such as anti-Smith (Sm) and anti-double stranded DNA (anti-dsDNA) are associated with the clinical presentation of the disease (18), and the level of anti-dsDNA frequently correlates with SLE disease activity (19).

Apart from the production of autoantibodies, B cells play additional roles in the pathogenesis of SLE. In lupus prone mice, B cells that do not secrete autoantibodies are still important to disease progression (20). This indicates that other B cell functions, such as antigen presentation to T cells may be of importance. Furthermore, B cells display hyperactivity in SLE (21), as well as increased expression of several toll-like receptors (TLRs) compared with healthy individuals (22), which may contribute to the inflammatory state. Thus, B cells are important players in several aspects of the SLE pathogenesis, and reducing the stimulation and numbers of B cells has been an important part of drug research.

B cells initially develop in the fetal liver and adult bone marrow and can be characterized by the use of surface markers such as CD19, CD20 and CD22, expressed at different stages of maturation. The development and survival of B cells depend upon stimulation by the B cell activating factor belonging to the tumor necrosis factor (TNF) family (BAFF), also known as B lymphocyte stimulator (BLyS). BAFF is a member of the TNF ligand superfamily of proteins, and is mainly produced by myeloid and stromal cells (23). Stimulation with BAFF improves B cell survival, proliferation, and antibody production through binding to three known receptors expressed in B cells at different stages of maturation, i.e., the BAFF-Receptor (BAFF-R; also known as BLyS receptor 3, BR3), transmembrane activator and calcium modulator and cyclophilin ligand interactor (TACI), and B cell maturation antigen (BCMA). BAFF transgenic mice develop symptoms characteristic of SLE (24), and BAFF levels are increased in patients with SLE compared with healthy controls and correlate with disease activity (25–28). In addition to BAFF, B cells are stimulated by cytokines such as a proliferation-inducing ligand (APRIL), which mainly serves as a plasma cell survival factor, interleukin (IL)-6, IL-21 and type I interferons (IFNs).

**Figure 1:**
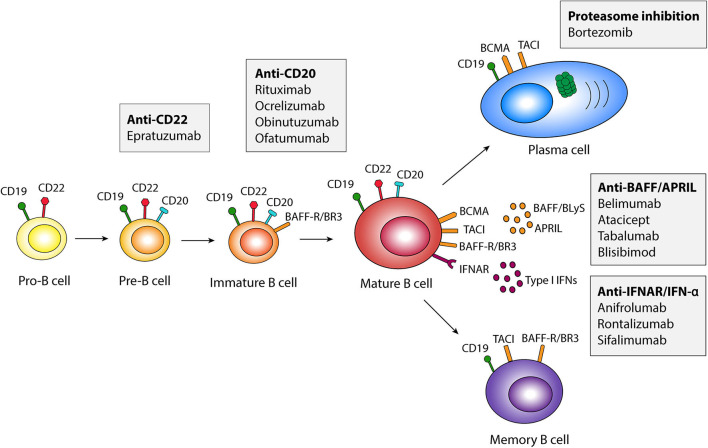
Schematic illustration of pharmaceuticals targeting B cells in different developmental stages. APRIL, a proliferation-inducing ligand; BAFF, B cell activating factor belonging to the tumor necrosis factor family; BAFF-R, BAFF Receptor; BCMA, B cell maturation antigen; BLyS, B lymphocyte activator; BR3, BLyS receptor 3; IFN, interferon; IFNAR, type I IFN receptor; TACI, transmembrane activator and calcium modulator and cyclophilin ligand interactor.

To inhibit B cell responses in SLE, two main pathways are currently used, i.e., (i) BAFF inhibition, and (ii) B cell depletion targeting the cell surface receptor CD20. The BAFF inhibitor belimumab was the first biological medication approved in 2011 by the US Food and Drug Administration (FDA) and the European Medicines Agency (EMA) for use in SLE. Belimumab is a recombinant human IgG1-λ monoclonal antibody that inhibits the soluble form of BAFF, preventing its interaction with BAFF receptors, thus inhibiting B cell survival and maturation. In contrast, rituximab is a chimeric anti-CD20 IgG1 monoclonal antibody that targets the CD20 molecule on the surface of B cells. This leads to B cell depletion through apoptosis, antibody dependent cell mediated cytotoxicity (ADCC), or antibody-dependent phagocytosis (ADP). Pharmaceuticals directly and indirectly targeting B cells that are used or have been trialed in SLE are illustrated in Figure 1, and summarized in Table 1.

**Table 1:** Pharmaceuticals with direct or indirect impact on B cells currently used or trialed for systemic lupus erythematosus.

**Drug name**	**Mechanism of action**	**Phase**	**Main results**	**References**
**B cell depleting agents**			
Epratuzumab	Humanized anti-CD22	III	Primary endpoint not met	Clowse et al. (55)
Obinutuzumab	Humanized anti-CD20	II	Primary and secondary endpoints met	Furie et al. (56)
Ocrelizumab	Humanized anti-CD20	III	Primary endpoint not met	Mysler et al. (53)
Ofatumumab	Fully human anti-CD20	R-L	Well-tolerated; reduced proteinuria	Haarhaus et al. (58)
		R-L	Well-tolerated; safe; efficacy implied	Masoud et al. (59)
Rituximab	Chimeric anti-CD20	II/III	Primary and secondary endpoints not met	Merrill et al. (30)
		III	Primary endpoint not met	Rovin et al. (31)
**B cell survival factor inhibitors**			
Atacicept	Blocks BAFF and APRIL	II/III	Serious infections; terminated	Ginzler et al. (89)
Belimumab	Fully human anti-BAFF	III	Superiority over placebo	Navarra et al. (10)
		III	Superiority over placebo	Furie et al. (11)
		III	Superiority over placebo	Stohl et al. (73)
		III	Superiority over placebo	Zhang et al. (72)
		III/IV	Primary endpoint not met	D'Cruz et al. (77)
Blisibimod	Inhibits soluble and membrane-bound BAFF	IIb	200 mg weekly superior over placebo	Furie et al. (90)
		III	Primary endpoint not met	Merrill et al. (91)
Tabalumab	Human monoclonal antibody binding soluble and membrane-bound BAFF	III	Primary endpoint not met	Isenberg et al. (92)
		III	120 mg every 2 weeks superior over placebo	Merrill et al. (93)
**Terminal stage B cell immunomodulators**			
Bortezomib	Proteasome inhibitor	II	Frequent adverse reactions	Ishii et al. (109)
		R-L	Efficacy implied	Alexander et al. (107)
		R-L	Efficacy implied	Sjöwall et al. (108)
**B cell depletion and survival factor inhibition combined**			
Rituximab and belimumab	Chimeric anti-CD20 and fully human anti-BAFF	II	Recruitment completed	Jones et al. (115)
		III	Recruitment completed	Teng et al. (116)
		II	No benefit of add-on belimumab to rituximab and	Aranow et al. (117)
			cyclophosphamide; LN	
		IIa	NET formation reduced; LN	Kraaij et al. (118)
		II	Recruiting; LN	NCT03747159
**Agents with indirect impact on B cells**			
Anifrolumab	Fully human anti-IFNAR	III	Primary endpoint not met	Furie et al. (126)
		III	Superiority over placebo	Morand et al. (127)
Rontalizumab	Humanized anti-IFN-α	II	Primary endpoint not met	Kalunian et al. (124)
Sifalimumab	Fully human anti-IFN-α	IIb	Superiority over placebo	Khamashta et al. (123)

*This table summarizes key clinical trials and observational studies of pharmaceuticals used or trialed for systemic lupus erythematosus, which directly or indirectly impact on B cells. Observational real-life studies are provided when clinical trial data are not available or scarce*.

*APRIL, a proliferation-inducing ligand; BAFF, B cell activating factor belonging to the tumor necrosis factor family; IFN, interferon; IFNAR, type I IFN receptor; LN, lupus nephritis; NET, neutrophil extracellular trap; R-L, real-life*.

## B Cell Depleting Therapies

### The Rationale for Rituximab

The chimeric anti-CD20 monoclonal antibody rituximab was approved by the FDA in 2006 for use in RA, and has been used off-label in the treatment of refractory SLE (29). The initial uncontrolled studies of rituximab in SLE showed encouraging results with improvements in both the clinical and laboratory compartment of the disease. However, two phase III randomized controlled trials have been performed, the EXPLORER trial in non-renal SLE (30) and the LUNAR trial in renal disease (31), none of which met their primary endpoints of significant reduction of disease activity compared with placebo (32).

### The Clinical Trial Failures

Based on experience from rheumatoid arthritis (RA), the most commonly used regimen for rituximab in clinical practice consists of two intravenous infusions of 1,000 mg each, given 14–21 days apart. In the EXPLORER study, 257 patients with moderate to severe non-renal SLE were randomized to receive rituximab or placebo. Rituximab in EXPLORER was administered at a dose of 1,000 mg at week 0, 2, 24, and 26 on a background of azathioprine, methotrexate, or mycophenolic acid therapy. At week 52, there was no difference between the active treatment and placebo groups in the primary endpoints (30), which comprised achievement and maintenance of a major, partial or no clinical response assessed using the eight British Isles Lupus Assessment Group (BILAG) index organ system scores (33). Nonetheless, in a subgroup analysis, rituximab showed benefit over placebo regarding major clinical response in African-American and Hispanic patients (30). In the LUNAR trial, 144 patients with class III or IV lupus nephritis on mycophenolic acid were randomized to receive placebo or rituximab, again at a dose of 1,000 mg at weeks 0, 2, 24 and 26. Also in this study, rituximab failed to achieve the primary endpoint, and there was no significant difference between the placebo and treatment arms regarding the proportion of patients who achieved complete or partial renal response (31). Afterwards, concerns have been raised regarding the concomitant use of high doses of glucocorticoids and immunosuppressive therapy in the EXPLORER and LUNAR trials, potentially clouding the effect exerted by rituximab. Several other factors may have played roles in the disappointing results of these trials, including inappropriate endpoints, the size of study populations and patient heterogeneity (32).

### The Promising Reports From Real-Life Use

Despite the negative clinical trials, the European League Against Rheumatism (EULAR) recommendations for the management of SLE prompt consideration of rituximab for organ-threatening SLE that has been refractory or shown intolerance to standard of care immunosuppressants (4). Moreover, the joint EULAR/European Renal Association—European Dialysis and Transplant Association (ERA-EDTA) recommendations for the management of lupus nephritis (34) and the American College of Rheumatology (ACR) guidelines for the management of renal SLE (35) recommend the use of rituximab as a rescue treatment in active renal SLE that has been non-responsive to standard therapy.

Indeed, targeting CD20 with rituximab has been endorsed in several centers where it is used as an off-label therapeutic option in SLE, mostly for refractory renal disease, either alone or as an add-on treatment to cyclophosphamide or mycophenolic acid (36–43), but also for other organ manifestations when conventional treatment has failed, e.g., severe lupus polyarthritis, hematological aberrancies and neuropsychiatric lupus (43–48). However, the use of rituximab has also raised some concerns regarding untoward effects, such as infusion-related reactions (49–51) and an increased frequency of post-rituximab late-onset neutropenia in SLE compared with other diseases, which calls for an attentive surveillance of rituximab-treated patients (52).

### B Cell Depleting Therapies Other Than Rituximab

Besides rituximab, some additional biological therapies targeting B cells have been trialed in SLE. The anti-CD20 humanized monoclonal antibody ocrelizumab was evaluated in a phase III trial which included 381 cases with severe lupus nephritis. However, the trial was terminated early due to an imbalance in serious infections in the treatment arm, and ocrelizumab has not been studied further (53). Epratuzumab is a humanized monoclonal antibody directed against CD22, which was well-tolerated and yielded encouraging results in a phase IIb study, with an evident superiority of epratuzumab 2,400 mg monthly in inducing BICLA response compared with placebo (12, 54). Unfortunately, none of the two subsequent phase III trials of epratuzumab in lupus were able to show improvements in response frequencies when compared with placebo (55).

Obinutuzumab is another humanized anti-CD20 monoclonal antibody with superior B cell cytotoxic effects over rituximab implicated for patients with RA and SLE. This drug has been studied in a phase II clinical trial of lupus nephritis (NOBILITY; NCT02550652), designed to evaluate the safety and efficacy of the type II anti-CD20 monoclonal antibody obinutuzumab in patients with proliferative kidney disease. The first results were reported in the form of a conference abstract, where greater frequencies of complete and partial renal response were observed among patients who received obinutuzumab vs. placebo, both as an add-on to mycophenolate mofetil and glucorticoids (56). Finally, the fully human monoclonal antibody ofatumumab, approved for the treatment of chronic lymphocytic leukemia, has shown encouraging results in smaller groups of patients with lupus manifestations such as autoimmune hemolytic anemia, immune-mediated thrombocytopenia and lupus nephritis (57, 58). These last two agents could be of particular interest for patients in whom rituximab has shown efficacy but infusion reactions have prompted discontinuation (59), or patients who did not achieve complete B cell depletion following treatment with rituximab (50).

## Inhibition of B Cell Survival Factors

### Rationale

Due to its important role in B cell homeostasis, BAFF has been of central interest as a target molecule in B cell pharmacotherapy in SLE. Belimumab, formerly known as Lympho-Stat B, was the first drug to be licensed for SLE in more than 60 years, and is still the sole biological agent approved for use in adult SLE since 2011 and pediatric and adolescent SLE since 2019. The efficacy of belimumab in reducing lupus activity was first shown in two phase III randomized, placebo-controlled clinical trials (10, 11), and patients with serological activity, high BAFF levels, low baseline B cell counts, limited or no organ damage and no exposure to tobacco were later demonstrated to be more benefited (60–67). Belimumab is a recombinant human IgG1-λ monoclonal antibody that specifically binds to the soluble form of BAFF. Normally, the binding of BAFF to B cells prolongs their survival and promotes their maturation and differentiation toward immunoglobulin and autoantibody production (68). BAFF signaling also leads to increases in anti-apoptotic proteins (69). As defective clearance of apoptotic cells is implicated in the pathogenesis of SLE and stimulation of autoantibody production, reductions in anti-apoptotic proteins upon BAFF inhibition may be expected to hamper this B cell-driven component of lupus pathogenesis.

### Clinical Trials and Observational Studies of Belimumab

Early trials of belimumab in SLE were inconclusive. A phase II trial that comprised 449 patients failed to meet its primary endpoints (9). However, a significant proportion of study participants (30%) had no elevated titres of antinuclear antibodies (ANA) at baseline, and the validity of their diagnosis was later questioned. To this point, it is important to mention that ANA have been shown to be less common than generally assumed in established cases of SLE (70, 71), which still is a matter of debate.

The first successful randomized controlled trial of belimumab in SLE was the BLISS-52 trial. BLISS-52 comprised 865 patients with a moderate to severe SLE and positivity for immunological markers. Modest but consistent improvements through week 52 were displayed in patients who received belimumab across various clinical outcomes, and the trial met its primary endpoint, i.e., a significantly greater proportion of patients who received belimumab 10 mg/kg at week 0, 2, 4 and thereafter every fourth week met the SRI-4 criteria for response compared with placebo (10). A second phase III clinical trial of similar design, the BLISS-76 trial, comprised 819 patients. The main difference compared with BLISS-52 was that the observation period in BLISS-76 was prolonged to a total of 76 weeks. The primary efficacy endpoint was the same as that in BLISS-52, and was set to the evaluation visit of week 52. Although this endpoint was reached at week 52 with belimumab 10 mg/kg resulting in a greater proportion of SRI-4 responders than placebo, the results of the subsequent study period until week 76 were rather inconclusive (11). Since then, three more phase III trials have been performed. One assessed belimumab efficacy in a North East Asian SLE population (72), and another one assessed the efficacy of subcutaneous administration (73, 74); both reached their primary endpoint, i.e., SRI-4 response frequency at week 52. Another phase III/IV trial assessed the efficacy of belimumab in SLE patients of black race (EMBRACE) using the same primary endpoint, however with a modification in the SLE Disease Activity Index (SLEDAI) assessment for the proteinuria item to meet the SLEDAI-2K standard (75), as compared with scoring according to Safety of Estrogens in Lupus Erythematosus National Assessment (SELENA)-SLEDAI (76) in the original SRI (8). While the primary endpoint of EMBRACE was not achieved, patients with high disease activity were benefited (77). Finally, reports from several real-life clinical settings have confirmed clinical efficacy and steroid-sparing effects (61, 78–84).

The BLISS trials of belimumab excluded patients with severe active lupus nephritis, but a large proportion of study participants had a history of renal involvement and low to moderate proteinuria at the time of inclusion (10, 11). A *post-hoc* analysis demonstrated that these patients benefited from belimumab with regard to several organ-specific aspects, including rates of renal flares (85). A phase III randomized controlled trial has been designed to specifically assess the effect of belimumab as an add-on to standard of care therapy in patients with active renal SLE, i.e., the BLISS-LN trial (NCT01639339), and publication of the first results is awaited. In a recent press release, the pharmaceutical company announced that BLISS-LN met its primary and key secondary endpoints (86), which paves the way for increasing use of B cell-targeted immunomodulation in this severe lupus manifestation (87).

### B Cell Survival Factor Inhibitors Other Than Belimumab

Atacicept is another BAFF-blocking biological agent that has been studied as a candidate pharmaceutical for SLE. Being a receptor construct that combines TACI with the Fc portion of human IgG, atacicept blocks the effects of both BAFF and its homologous B cell cytokine APRIL (88). Unfortunately, a clinical trial of atacicept in lupus nephritis was prematurely terminated due to adverse events in the form of hypogammaglobulinemia and infections (89), but attempts with adjusted dosing have not been totally abandoned.

Blisibimod is a fusion protein consisting of four high-affinity BAFF-binding domains and the Fc domain of human IgG1, and targets both soluble and membrane-bound BAFF. A dose-ranging phase IIb clinical trial (90) determined a safe and effective dose of blisibimob to be further studied in a subsequent phase III clinical trial, which however failed to meet its primary endpoint (91).

Only one of the two phase III clinical trials of tabalumab, a fully human monoclonal antibody that targets soluble and membrane-bound BAFF, met its primary endpoint, i.e., proportion of patients achieving SRI-5 at week 52 (92, 93), and no further development of this drug was therefore planned for SLE. However, it is worth noting that no dose-ranging phase II studies had preceded the phase III trials. Several key outcomes in both trials still justify the rationale of targeting both the cleaved and membrane-bound BAFF counterparts (94, 95).

## Modulating the Terminal Maturation Stage of B Cells

### The Rationale for Proteasome Inhibition

The majority of the immunosuppressants used in SLE exert their therapeutic effects on B cells, plasmablasts and short-lived plasma cells (96). However, to achieve effects beyond this, i.e., on the long-lived plasma cells, the only available alternatives are autologous stem cell transplantation, atacicept (blocking both BAFF and APRIL) and proteasome inhibition (97–99). This was the rationale for using bortezomib in SLE cases resistant to conventional therapy.

Bortezomib is a specific, reversible, and cell permeable dipeptide boronic acid inhibitor of the chymotryptic activity of the 20S subunit of the proteasome, approved for the treatment of multiple myeloma and mantle cell lymphoma (100). Proteasome inhibition causes accumulation of defective immunoglobulin chains, resulting in endoplasmic reticulum stress, misfolded protein response, and subsequent apoptosis of plasma cells (101, 102). In addition, the long-lived plasma cells are vigorous antibody producers, and are thus highly sensitive to proteasome inhibition (99). On the other hand, proteasome inhibitors also effectively function as inhibitors of the production of pro-inflammatory cytokines through the regulation of NF-κB activation (103). Promising results in experimental lupus models and reports on use of bortezomib for allograft rejection in kidney transplantation (104, 105) have given rise to the concept of using bortezomib for patients with refractory lupus (106).

### Evidence From Clinical Trials and Observational Studies

Several cases with refractory and life-threating manifestations of SLE in Germany and Sweden were treated with bortezomib and encouraging results were reported (107, 108). In a recent Japanese multicentre double-blind randomized controlled phase II trial, which enrolled 14 patients with persistently raised disease activity, patients were randomized to receive either bortezomib as an add-on therapy to their concomitant immunosuppressants or placebo (109). Unfortunately, albeit obvious clinical efficacy was seen in several patients, some of the patients who received bortezomib experienced adverse reactions, i.e., fever, severe hypersensitivity, or other infusion reactions. The authors recommended to carefully select patients for bortezomib therapy, and use protocols to prevent side-effects.

## Combining B Cell Therapies

### Rationale

Since rituximab induces B cell depletion, but also results in elevation of BAFF levels, studies have examined whether the increased BAFF levels may promote re-expansion of autoreactive B cells and by extension an earlier relapse. The effects of rituximab are dependent on the degree of B cell depletion, and incomplete depletion has been shown to be associated with lower frequencies of clinical response (27). In patients with refractory SLE with high levels of anti-dsDNA antibodies, relapse occurred at lower B cell numbers, and plasmablasts represented a larger percentage of the B cell population (110). Following rituximab administration, levels of BAFF rise (111), and BAFF levels are higher at relapse after rituximab treatment compared with disease flare before rituximab treatment (112). Further, quantifiable BAFF in serum has been associated with shorter clinical response to rituximab in patients with refractory SLE (113). Thus, a contributing factor to the lack of efficacy of rituximab in randomized clinical trials may be the increased BAFF levels following rituximab administration. Theoretically, combining rituximab with belimumab could give a more thorough and sustained inhibition of B cell responses, as speculated in early investigations (28, 111, 112, 114). This is currently evaluated in several clinical trials, e.g., BEAT Lupus (115) and BLISS-BELIEVE (116).

It is of particular importance that the merit of combining B cell therapies has also been conceptualized in the context of lupus nephritis. The Rituximab and Belimumab for Lupus Nephritis (CALIBRATE; NCT02260934) (117) and the investigator-initiated Synergetic B cell Immunomodulation in SLE (SynBioSe) trials (SynBioSe 1: NCT02284984; SynBioSe 2: NCT03747159) were designed to assess the efficacy of rituximab and belimumab combined in active lupus nephritis. The proof-of-concept open label SynBioSe 1 is completed, and a first report demonstrated reductions in antinuclear antibodies and neutrophil extracellular trap (NET) formation (118). SynBioSe 2 is currently recruiting, and results may be anticipated by the end of 2023.

## Perspective: Future Ways of Targeting B Cells

Autoreactive B cells are indubitably key cells in the pathogenesis of SLE, but the theoretical merit has hitherto seldom culminated in the anticipated outcomes in drug development. The lack of success in clinical trials has not been for lack of trying. Apart from pharmaceuticals which predominantly exert effects on B cells, numerous other therapeutic modalities have been trialed for SLE, several of them expected to indirectly impact on B cells and B cell functions. For example, in lupus prone mice, targeting other B cell stimulating cytokines, such as IL-6, decreased disease progression, but this strategy did not succeed in subsequent clinical trials (119). Targeting the co-stimulatory molecule CD40 led to modest clinical improvement, but also unacceptable side-effects in the form of thromboembolic events (120).

Activation of the type I IFN pathway is prominent in the pathogenesis of SLE, and type I IFNs stimulate BAFF production. In patients with SLE, the type I IFN pathway is overexpressed, and the IFN-α protein in particular has shown associations with both disease activity (121) and risk of relapse (122). IFNs are pleiotropic cytokines with numerous functions in the immune response equilibrium, including an impact on B cells. Thus, albeit not exclusive, the effects of IFN inhibition are attractive also in the B cell context.

The first reports to support the efficacy of direct IFN-α inhibition in SLE originated from a phase IIb clinical trial of sifalimumab (123). The results were modest, but in favor of sifalimumab. Unfortunately, a phase II trial of the anti-INF-α rontalizumab demonstrated that rontalizumab was superior over placebo in SLE patients with low IFN-regulated gene expression, but not in patients with high IFN gene signature (124), contrary to what expected considering its biologic mechanism.

Following promising results in a phase II clinical trial (125), the type I IFN receptor (IFNAR) inhibitor anifrolumab was evaluated in two phase III trials, i.e., TULIP-1 and TULIP-2. In TULIP-1, the primary outcome, i.e., SRI-4 response, was not met (126). By contrast, a greater proportion of patients receiving anifrolumab vs. placebo in TULIP-2 met the primary outcome, i.e., BICLA (127). Possible reasons for the discrepancy between the TULIP trials may include the choice of outcomes and the study populations. The primary endpoint in TULIP-2 was initially planned to be SRI-4. However, this was changed at a later stage, upon a subanalysis of TULIP-1 where proportions of BICLA unlike SRI-4 responders favored anifrolumab. Notably, in TULIP-2 both SRI-4 and BICLA showed ability to separate treatment arms.

An interesting trend is targeting B cell intracellular signaling, such as through inhibition of Bruton's Tyrosine Kinase (BTK), which is a strategy approved for the treatment of B cell malignancies. Inhibition of BTK has shown efficacy in lupus prone mice, which resulted in reduced kidney damage and increased survival (128). Another development originating in the area of cancer therapy was the chimeric auto-antigen receptor (CAAR) T cells. CAAR-T cells have been genetically engineered to kill human autoreactive B cells specific toward desmoglein-3 in pemphigus vulgaris (129), and in two lupus mice models, use of CAAR-T cells targeting the CD19 surface molecule resulted in reduced kidney damage and increased survival (130). Although long-term data are not available, evidence suggests that the CAAR-T cells acquire a long-term memory phenotype and persist in peripheral tissue of patients.

### Epilog

To summarize, B cell hyperactivity and breach of tolerance constitute hallmarks of SLE, and it is widely accepted that B cells play central roles in the pathogenesis. However, the contribution of B cells to disease initiation and perpetuation is less well understood. B cells in SLE constitute the main autoantibody producers and probably facilitate the priming of autoreactive T cells and function as antigen-presenting cells, as well as constitute a source of the cytokines involved in immune dysregulation (131). As a result, many of the therapeutic agents that have been trialed in SLE target B cell-related pathways.

Even though drug development in the field of SLE has been slow, B cell-targeting therapies have been increasingly used during the last two decades and contributed to improved management and improved prognosis. The amount and primary results of ongoing clinical trials prophesy that B cell therapies of selective targets will have an established place in the future personalized therapeutic management of lupus patients.

## Author Contributions

All authors contributed to the manuscript draft, critically reviewed all parts of the manuscript, accepted its final version prior to submission, and account for its content.

## Conflict of Interest

The authors declare that the research was conducted in the absence of any commercial or financial relationships that could be construed as a potential conflict of interest.
